# Editorial: Neurobiological, psychological, and environmental influences on parenting and child development: an inclusive and interdisciplinary perspective

**DOI:** 10.3389/fpsyg.2025.1676172

**Published:** 2025-08-29

**Authors:** Michele Giannotti, Paola Rigo, Nicola Carone, Karen Hazell Raine

**Affiliations:** ^1^Universita degli Studi di Trento Dipartimento di Psicologia e Scienze Cognitive, Rovereto, Italy; ^2^Universita degli Studi di Padova Dipartimento di Psicologia dello Sviluppo e della Socializzazione, Padua, Italy; ^3^Universita degli Studi di Roma Tor Vergata Dipartimento di Medicina dei Sistemi, Rome, Italy; ^4^School of Nursing and Midwifery, University of Newcastle, Newcastle, NSW, Australia

**Keywords:** parenting, child development, family diversity, caregiving, socio-cultural factors

Parenting is a complex, multifaceted construct emerging from the dynamic interplay of neurobiological, psychological, and socio-cultural factors. A substantial body of literature has highlighted parenting's central role in fostering child development across emotional, cognitive, social, and physical domains (Bornstein, 2019). Therefore, understanding the underlying processes that determine maternal and paternal caregiving behaviors is essential. Accumulating evidence underscores that integrating these dimensions through interdisciplinary, multi-method approaches is crucial to moving beyond the nature vs. nurture dichotomy and to account for the diversity of contemporary family structures, including increased father involvement, and the challenges and opportunities faced by contemporary families (Carone et al., 2021; Giannotti et al., 2022).

Considering this complexity, the goal of this Research Topic is to offer a broad and inclusive perspective on parenting by adopting an interdisciplinary framework. This approach includes deepening the understanding of predictors, correlates, and outcomes of caregiving in diverse family forms such as single-parent, same-sex, different-sex, adoptive, and foster families, across both clinical and non-clinical contexts (Jensen and Sanner, 2021). The topic aims to bridge disciplines by encompassing theoretical and empirical contributions that integrate neurobiological, psychological, and socio-cultural perspectives. Taken together, these findings highlight the importance of interdisciplinary approaches to understanding parenting and emphasize the need to consider the diversity of family structures and cultural contexts when designing preventive strategies and interventions. Ultimately, this Research Topic aims to stimulate debate and further research on the complex mechanisms shaping parenting quality and child development, promoting inclusive policies that support and target common and specific needs of diverse family forms. This editorial presents a summary of the main results for the 11 manuscripts included in this Research Topic.

Focusing on cultural influences, Pickron and Kutlu found that multiracial and multilingual infants may develop greater perceptual flexibility than those exposed to only one domain. Multilingual infants exhibit reduced racial bias, and combining linguistic and racial diversity may enhance perceptual plasticity. Inclusive research in multiethnic and multidialectal settings can thus improve reproducibility and deepen our understanding of sociocognitive development. In the cross-cultural domain, motherhood experiences vary widely due to exclusion, discrimination, and poverty. Among marginalized Roma communities, parenting behaviors, especially harsh discipline and low stimulation, are shaped by maternal education, poverty level, and stress. Poverty partially mediates stimulation differences. These insights can inform targeted early interventions and shift public discourse (Van Laer et al.).

At the environmental and relational level, the role of early caregiving is underscored in studies exploring both disruption and adaptation. One study examined children adopted after early maternal separation and brief institutional or foster care, finding elevated dissociative symptoms and behavioral difficulties despite stable post-adoption environments, revealing long-lasting effects of early relational adversity on socioemotional functioning (Winnette et al.). These findings emphasize how even time-limited disruptions in sensitive caregiving during infancy may leave enduring psychological imprints.

In contrast, a pilot intervention demonstrated how maternal adaptation through bodily-tactile communication significantly enhanced emotional availability in mother-child dyads where the child had visual impairment and additional disabilities, illustrating how compensatory sensory strategies can foster connection despite profound challenges (Peltokorpi et al.). Mothers increased their use of tactile signals and anticipatory cues, which in turn improved child engagement and emotional responsiveness, highlighting the transformative potential of touch-based interaction in complex developmental contexts.

Next, focusing on early childhood, a study of Italian families with 8-year-olds examined parenting's impact on prosocial and externalizing behaviors. Results showed that both same- and different-gender parent families displayed high warmth and low hostility. Positive parenting correlated with increased prosocial behavior and decreased externalizing behavior, regardless of parental gender, highlighting the importance of supportive parenting in children's behavioral adjustment (Baiocco et al.).

Meanwhile, parents of children with ADHD often struggle with effective strategies, leading to stress and behavioral issues. In Brazil, a randomized clinical trial compared online and face-to-face behavioral parent training (BPT) for ADHD and ODD. Both formats significantly reduced symptoms and improved quality of life; the online self-directed platform was equally effective, offering a scalable, affordable alternative (Paiva et al.).

In families coping with parental cancer diagnoses, affecting about 50,000 German children annually, a qualitative study using the Family Resilience Framework identified how families adapt: via social support, cohesion, adaptability, clear communication, psychological well-being, financial stability, and proactive information-seeking. Spirituality and collaborative problem-solving were less influential, suggesting directions for clinical guidance (Heuser et al.).

Finally, sensory integration training (SIT) has proven effective for children with autism spectrum disorder, enhancing balance and executive function. Tools like Footscan (for biomechanics) and fNIRS (for neural activation) show how SIT addresses sensory-processing deficits in vision, vestibular perception, and proprioception. This multidisciplinary approach underscores links between sensory-motor function and cognitive control, indicating integrated therapeutic potential, though more research is needed to clarify mechanisms (Deng et al.).

Psychologically, children's temperamental traits and parental perceptions are also central. A cluster analysis of parent-teacher reports of behavioral inhibition revealed that discrepancies are not mere noise but convey domain-specific insights into children's internalizing difficulties, with parenting behaviors such as hostility and lax or physical control predicting high behavioral inhibition, particularly in boys (Sulyok et al.). These patterns reinforce the importance of multi-informant assessments and gender-sensitive interventions. Similarly, a large-scale study on Chinese adolescents found that parenting marked by care and autonomy support protected against depression, while control increased vulnerability, effects that were mediated by adolescents' self-compassion, pointing to a promising psychological buffer against parental risk (Ren et al.). Finally, contextual factors such as paternal education and parenting style also shape academic adjustment. Fathers' overparenting mediated the relationship between their educational level and children's school problems, with child gender and surgency moderating outcomes, reinforcing the need for nuanced, individualized perspectives on father–child dynamics (Ruiz-Ortiz et al.).

Taken together, this Research Topic demonstrates that effective parenting emerges from complex neurobiological, psychological, and environmental interactions across diverse family structures. The interdisciplinary findings underscore the need for inclusive, culturally-sensitive interventions that recognize varied pathways through optimal child development while supporting all families' unique strengths and challenges.

## References

[B1] Bornstein M. H. (2019). “Parenting infants,” in Handbook of Parenting, 3rd Edn, ed. Bornstein M. H. (New York: Routledge), 3–55.

[B2] Carone N. Bos H. Shenkman G. Tasker F. (2021). Editorial: LGBTQ parents and their children during the family life cycle. Front. Psychol. 12:643647. 10.3389/fpsyg.2021.643647 33679568 PMC7930207

[B3] Giannotti M. Gemignani M. Rigo P. Venuti P. De Falco S. (2022). The role of paternal involvement on behavioral sensitive responses and neurobiological activations in fathers: a systematic review. Front. Behav. Neurosci. 16:820884. 10.3389/fnbeh.2022.820884 35355925 PMC8959913

[B4] Jensen T. M. Sanner C. (2021). A scoping review of research on well-being across diverse family structures: rethinking approaches for understanding contemporary families. J. Family Theor. Rev. 13, 463–495. 10.1111/jftr.12418

